# E2-25K SUMOylation inhibits proteasome for cell death during cerebral ischemia/reperfusion

**DOI:** 10.1038/cddis.2016.428

**Published:** 2016-12-29

**Authors:** Eun Il Jeong, Hae Won Chung, Won Jea Lee, Seo-Hyun Kim, Hyunjoo Kim, Seon-Guk Choi, Yong-Keun Jung

**Affiliations:** 1 Global Research Laboratory, Department of Biological Science, Seoul National University, 1 Gwanak-ro, Gwanak-gu, Seoul 151-747, Korea

## Abstract

Cerebral ischemia/reperfusion (I/R) causes brain damage accompanied by ubiquitin accumulation and impairment of proteasome activity. In this study, we report that E2-25K, an E2-conjugating enzyme, is SUMOylated during oxidative stress and regulates cerebral I/R-induced damage. Knockdown of E2-25K expression protects against oxygen/glucose deprivation and reoxygenation (OGD/R)-induced neuronal cell death, whereas ectopic expression of E2-25K stimulates it. Compared with the control mice, cerebral infarction lesions and behavioral/neurological disorders are ameliorated in E2-25K knockout mice during middle cerebral artery occlusion and reperfusion. In particular, E2-25K is SUMOylated at Lys14 under oxidative stress, OGD/R and I/R to prompt cell death. Further, E2-25K downregulates the proteasome subunit S5a to impair proteasome complex and thus restrain proteasome activity under oxidative stress. This proteasome inhibitory activity of E2-25K is dependent on its SUMOylation. These results suggest that E2-25K has a crucial role in oxidative stress and cerebral I/R-induced damage through inhibiting proteasome via its SUMOylation.

Stroke is a major cause of mortality and disability in humans. Because of the intricate pathological characteristics of ischemic neuronal cell death, diverse mechanisms and molecules associated with cerebral ischemia/reperfusion (I/R) have been reported. The mechanisms that are known to protect against I/R-induced neurotoxicity include: hypothermia,^1, 2^ autophagy^3^ (Tsc1),^4^ preconditioning^5, 6^ and activation of survival factors, such as HIF1,^7, 8^ Nrf2,^9^ EPO,^10^ BDNF^11^ and so on. In addition, cell death processes elicited by cerebral I/R include excitotoxicity (NMDAR),^12, 13^ ion imbalance (Ca^2+^),^14, 15^ inflammation^16, 17^ and oxidative stress.^18, 19^ Among them, an excess of reactive oxygen species (ROS) attributed to perturbation of mitochondrial metabolism, lipid peroxidation and inflammation response during I/R has a vital role in cell fate determination of the damaged neurons. Thus, understanding the ROS-mediated molecular events under I/R damage is important.

The ubiquitin–proteasome system is one of the main mechanisms for protein degradation. Short-lived or abnormal proteins are tagged by covalent modification of ubiquitin using E1, E2 and E3 enzymes.^20^ Target proteins labeled with ubiquitin are then recognized by large protein complexes, proteasomes. When the number of damaged proteins is too large to be quickly removed, it leads to aggregation in pathological conditions. In addition, the impaired proteasome activity can also induce the accumulation of aggregation-prone proteins and damaged proteins. Evidence from a number of studies suggests that the proteasome might have an important role in I/R^21, 22^ and cerebral I/R results in reduced proteasomal activity.^23^ Besides, I/R is also involved in immoderate production of various abnormal proteins due to oxidative stress and other mechanisms.^24^ These proteins are reflected in the prolonged accumulation of polyubiquitinated proteins that can be attributable to impaired proteasome and are observed in dying neurons but not in the remaining neurons that survive.^25^ However, the mechanism of inhibition of proteasome activity in neurons after I/R remains unknown.

E2-25K (also known as HIP2) is an ubiquitin-conjugating enzyme and is ubiquitously expressed with the highest level of expression in the brain.^26^ It is known to have a role in aggregate formation of expanded polyglutamine proteins and suppression of apoptosis in polyglutamine diseases, such as Huntington's disease.^27^ In Alzheimer's disease, E2-25K acts as a mediator of A*β* neurotoxicity, which is also accompanied by coordinating endoplasmic reticulum (ER) stress and caspase-12 activity.^28, 29^ Increasing evidence showed that E2-25K is also involved in the dislocation of newly synthesized MHC class I heavy chains from the ER,^30^ formation of foam cells^31^ and proteolysis of Rb induced by E7 in growth-arrested cells,^32^ thus indicating the diverse roles of E2-25K in many pathways.

In the present study, we observed that E2-25K was SUMOylated under oxidative stress and I/R to mediate neuronal cell death and brain injury. In this process, SUMOylated E2-25K was crucial for regulating proteasome activity through S5a.

## Results

### E2-25K mediates neuronal cell death under oxidative stress

**Figure 1:**
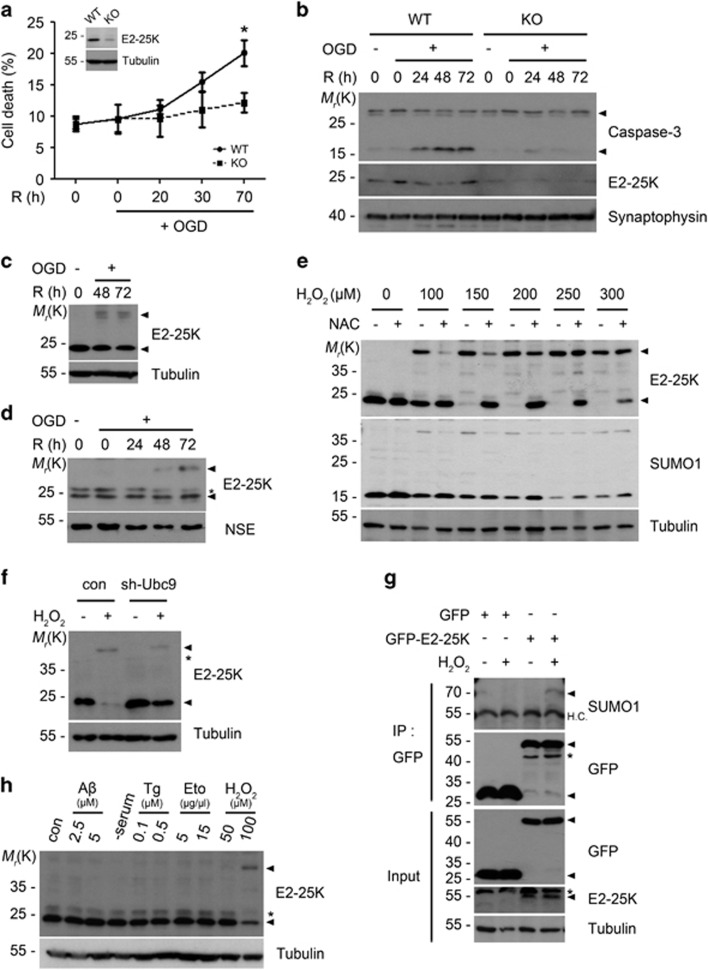
Neuronal cell death in OGD/R is blocked by E2-25K deficiency and accompanied by E2-25K SUMOylation. (**a** and **b**) E2-25K WT or KO mouse primary cortical neurons were cultured for 9 days *in vitro* (DIV-9) and exposed to OGD for 3 h followed by reoxygenation for the indicated times. Cell death rates were measured by trypan blue exclusion assay and expression level of E2-25K was examined by western blotting (insert). Values represent mean±S.E.M. (*n*=3, two-way ANOVA followed by Bonferroni's *post hoc* test, *P<0.05) (**a**). Cell extracts were examined with western blot analysis (**b**). (**c** and **d**) SH-SY5Y cells (**c**) and WT mouse primary cortical neurons (DIV-10) (**d**) were exposed to OGD for 3 h and reoxygenation for the indicated times. Cell extracts were then analyzed by western blotting. (**e**) SH-SY5Y cells were incubated with the indicated concentrations of H_2_O_2_ for 12 h in the presence or absence of preincubation with 500 *μ*M NAC for 1 h and analyzed by western blotting. (**f**) SH-SY5Y cells were transfected with Ubc9 shRNA for 24 h and treated with 150 *μ*M H_2_O_2_ for 22 h. (**g**) HEK293T cells were transfected with GFP or GFP-E2-25K for 24 h and then exposed to 150 *μ*M H_2_O_2_ for 18 h. Cell extracts were prepared and subjected to immunoprecipitation (IP) assay using anti-GFP antibody. The immunoprecipitates and cell extracts (input) were analyzed by western blotting. (**h**) SH-SY5Y cells were left untreated (con) or treated with A*β*1-42, thapsigargin (Tg), etoposide (Eto) or H_2_O_2_ for 16 h or serum-free medium (-serum) for 20 h

To characterize the role of E2-25K in I/R, we first examined the contribution of E2-25K to oxygen/glucose deprivation and reoxygenation (OGD/R)-induced neuronal cell death by targeting E2-25K expression with shRNA. We confirmed that E2-25K expression was abolished in B103/sh-E2-25K cells (Supplementary Figure S1a). Incubation of B103 control cells in OGD/R apparently induced cell death after 44 h (Supplementary Figure S1b), resulting in 69% cell death at 48 h (Supplementary Figure S1c) and activation of caspase-3 (Supplementary Figure S1d). Compared with control cells, B103/sh-E2-25K cells were significantly resistant to OGD/R-induced cell death (Supplementary Figures S1c and d). Similar results were observed in experiments performed with primary cultured mouse cortical neurons. E2-25K knockout (KO) neurons were less vulnerable to cell death (20% *versus* 12% at 70 h) than the wild-type (WT) neurons under OGD/R (Figure 1a). Accordingly, caspase-3 activation was suppressed in E2-25K KO cortical neurons (Figure 1b). Although E2-25K KO mice were previously generated by the gene-trap method,^29^ E2-25K expression was not completely abolished in cortical neurons. Further, knockdown of E2-25K expression in SH-SY5Y human neuroblastoma cells also resulted in increased cell viability under OGD/R (Supplementary Figure S1e). Conversely, overexpressed E2-25K significantly increased OGD/R-induced cell death. Similar to OGD/R, E2-25K was also critical for cell death triggered by H_2_O_2_ (Supplementary Figures S1f and g). Together, these results suggest that E2-25K mediates neuronal cell death under OGD/R and oxidative stress.

### E2-25K is SUMOylated only under oxidative stress

Interestingly, we observed that OGD/R induced higher molecular weight (38 kDa) of E2-25K, with in addition to its original form (24 kDa; Figures 1c and d). The appearance of 38 kDa E2-25K was proportional to the treated H_2_O_2_ concentration (100–300 *μ*M; Figures 1e and h), whereas 24 kDa E2-25K was concomitantly reduced. As expected, 38 kDa E2-25K was abolished and 24 kDa E2-25K was rescued by treatment with the antioxidant NAC (Figure 1e; Supplementary Figure S1h). FACS analysis showed that exposure to either OGD/R or H_2_O_2_ (either 100 or 200 *μ*M) generated similar levels of ROS in the cells (Supplementary Figure S1i).

Unlike in control cells, 38 kDa E2-25K was not detected in B103/sh-E2-25K cells even after exposure to H_2_O_2_ (Supplementary Figure S1j). In addition, 38 kDa E2-25K was proved as a SUMOylated form because it was also detected by anti-SUMO1 antibody (Figure 1e) and was reduced by knockdown of Ubc9, a SUMO-conjugating enzyme (Figure 1f). Further, immunoprecipitation (IP) analyses showed that GFP-fused E2-25K was SUMOylated in the transfected cells after exposure to H_2_O_2_ (Figure 1g). Unfortunately, there is no commercially available or no home-made antibody to perform IP assay for endogenous E2-25K. Therefore, we performed a semi-IP assay using GFP-fused E2-25K. All these results indicate that the 38 kDa band is SUMOylated E2-25K. Moreover, we found that such E2-25K SUMOylation occurred only under oxidative stress but not upon other toxic insults such as neurotoxic stress (A*β*), autophagy (serum deprivation), ER stress (thapsigargin) and apoptosis (etoposide) (Figure 1h).

### E2-25K SUMOylation at Lys14 promotes cell death

**Figure 2:**
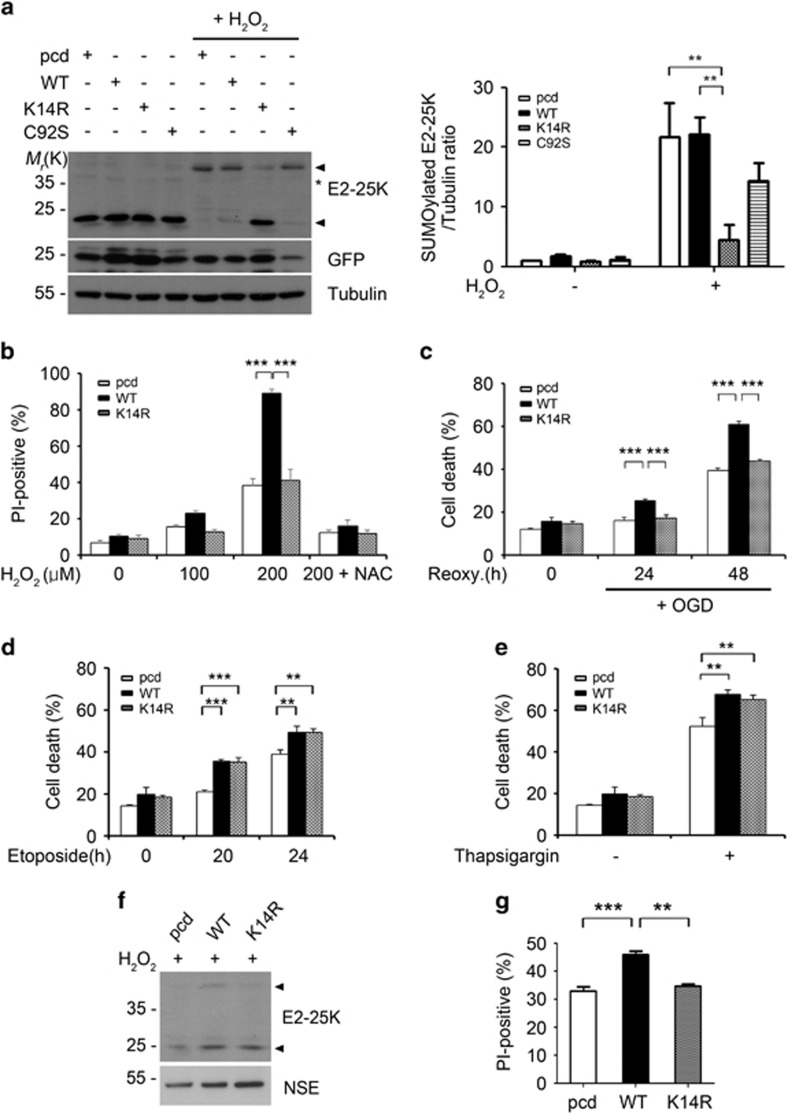
E2-25K SUMOylation at Lys14 promotes cell death under hypoxia and ROS stress. (**a**) SH-SY5Y cells were transfected with GFP (as a transfection efficiency control) and pcDNA (pcd), E2-25K WT or mutants (K14R, C92S) for 24 h and then treated with 150 *μ*M H_2_O_2_ for 9 h followed by western blotting (left). The signals on the blots were quantified and bars indicate mean±S.E.M. (*n*=3, two-way ANOVA followed by Bonferroni's *post hoc* test, **P<0.01; right). (**b**–**e**) SH-SY5Y were transfected with pcDNA (pcd), E2-25K WT or K14R for 24 h and exposed to H_2_O_2_ for 16 h (**b**), OGD/R (**c**), 50 *μ*g/*μ*l etoposide (**d**) or 2 *μ*M thapsigargin (**e**) for 24 h. Bars indicate mean±S.E.M. (*n*=3, two-way ANOVA followed by Bonferroni's *post hoc* test, **P<0.01. ***P<0.001). (**f** and **g**) E2-25K KO mouse cortical neurons (DIV-10) were transfected with pcDNA (pcd), E2-25K WT or K14R and treated with 100 *μ*M H_2_O_2_ for 12 h. The levels of SUMOylated E2-25K were examined by western blotting (**f**) and cell death rates were determined after propidium iodide staining (**g**) (mean±S.E.M., *n*=3, one-way ANOVA followed by Tukey's *post hoc* test, **P<0.01, ***P<0.001). Arrow heads and asterisks indicate E2-25K and non-specific signals, respectively

To evaluate the importance of E2-25K SUMOylation in oxidative stress-induced cell death, we utilized an E2-25K K14R in which the Lys14 residue was replaced with Arg. The E2-25K Lys14 was previously reported as a major SUMOylation site using an *in vitro* system.^34^ As predicted, SUMOylation of E2-25K K14R was blocked under oxidative stress and that of activity-dead E2-25K C92S was partially inhibited (Figure 2a). Utilizing these E2-25K mutants, we then assessed the contribution of SUMOylated E2-25K to cell death. H_2_O_2_-mediated cell death was augmented by E2-25K WT and partially by E2-25K C92S, but not by SUMOylation-defective E2-25K K14R (Figure 2b; Supplementary Figure S2). Likewise, increased cell death was observed under OGD/R by the overexpression of E2-25K WT, but not by E2-25K K14R (Figure 2c). On the other hand, these distinct effects of E2-25K WT and K14R on cell death under oxidative stress were not observed in cell death triggered by other toxic signals, such as etoposide (Figure 2d) or thapsigargin (Figure 2e). We further confirmed that H_2_O_2_-induced E2-25K SUMOylation (Figure 2f) and cell death (Figure 2g) were increased by reconstituting E2-25K KO cortical neurons with E2-25K WT but not by E2-25K K14R. Therefore, E2-25K SUMOylation at Lys14 selectively promotes H_2_O_2_- or OGD/R-mediated cell death.

### E2-25K deficiency improves MCAO/R injury-induced brain damage

**Figure 3:**
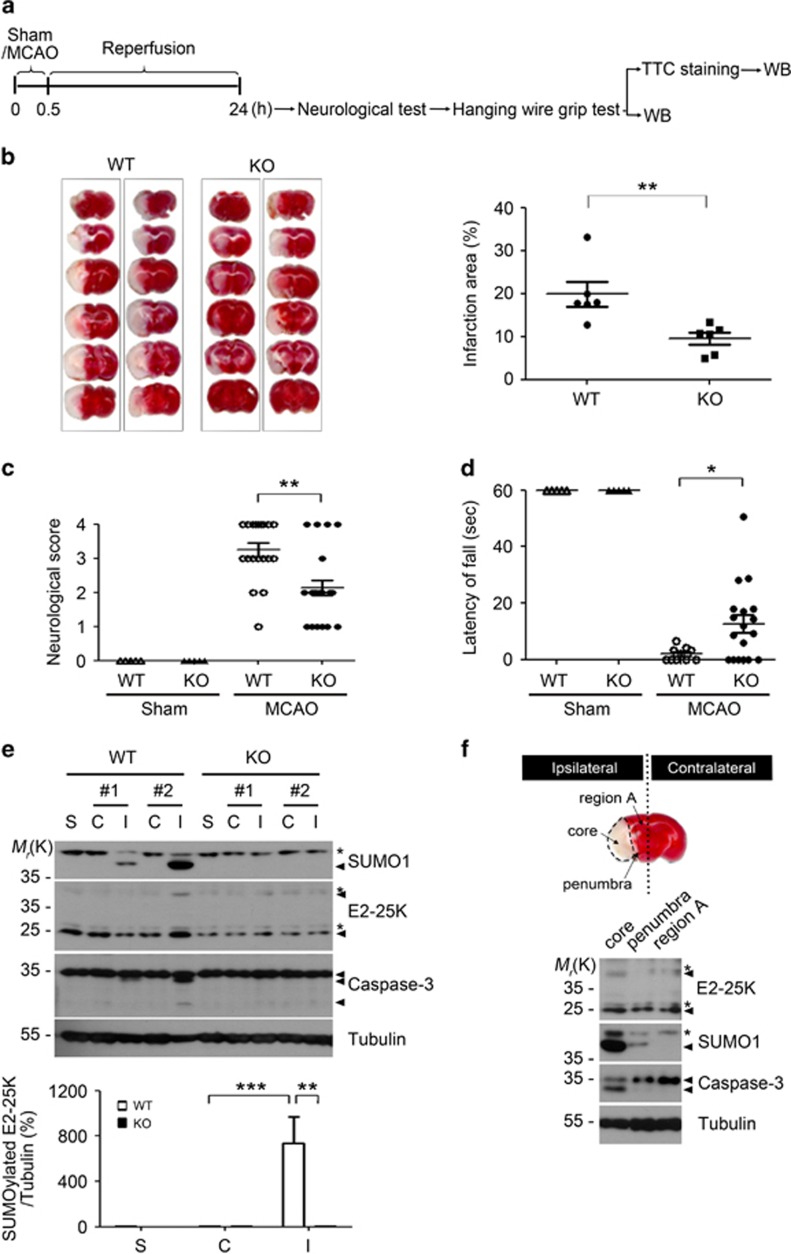
E2-25K deficiency ameliorates MCAO/R injury. (**a**) Schematic diagram showing the experimental schedule. (**b**) The 3–4-month-old male E2-25K WT and KO mice were subject to MCAO for 30 min and reperfusion for 24 h. The 2-mm coronal brain sections, which were prepared from the olfactory bulb to the cerebellum, were analyzed after TTC staining (*n*=2 for each genotype in this figure; left). Bars represent the percentage of infarction area to whole area with mean±S.E.M., WT mice, *n*=6; KO mice, *n*=8 for the analysis, unpaired two-tailed Student's *t*-test, **P<0.01 (right). (**c**) The neurological deficits in each mouse were assigned as a score (mean±S.E.M., each sham, *n*=5; WT mice, *n*=19; KO mice, *n*=22, two-way ANOVA followed by Bonferroni's *post hoc* test, **P<0.01). (**d**) The mice were analyzed with wire hanging test (mean±S.E.M., each sham, *n*=5; WT mice, *n*=10; KO mice, *n*=18, two-way ANOVA followed by Bonferroni's *post hoc* test, *P<0.05). (**e**) Brain extracts (except cerebellum) were prepared from non-ischemic (contralateral, C), ischemic (ipsilateral, I) and sham control (S) hemispheres of E2-25K WT and KO mice and analyzed by western blotting (upper). Bars indicate mean±S.E.M. (lower; *n*=4, two-way ANOVA followed by Bonferroni's *post hoc* test, **P<0.01, ***P<0.001). (**f**) TTC-stained section showing examples of lesions from MCAO/R-treated WT mouse. Brain extracts were prepared from ischemic core, penumbra and the rest of the region (region A) in the ipsilateral region (upper) and analyzed by western blotting (lower). Asterisks indicate non-specific signals

To examine the pathophysiological significance of E2-25K function and modification, we performed middle cerebral artery occlusion for 30 min followed by reperfusion for 24 h (MCAO/R) in WT and E2-25K KO mice (Figure 3a). 2,3,5-Triphenyltetrazolium chloride (TTC) staining revealed that the cerebral infarction lesion was significantly ameliorated (about two-fold) in E2-25K KO mice compared with that in WT mice (Figure 3b). Accordingly, E2-25K KO mice showed lower neurological scores than WT mice (Figure 3c). As cerebral I/R causes impairment of sensory-motor deficit and a decline in hanging ability, we additionally performed the hanging wire grip test to evaluate grip strength and endurance. Compared with WT mice, E2-25K KO mice showed remarkably improved grip strength after MCAO/R (Figure 3d). All these results indicate that E2-25K exhibits a detrimental effect on both cerebral I/R injury and behavioral neurological disorders.

We then characterized E2-25K-mediated I/R damage in the mouse brains. As observed in *in vitro* OGD/R assay, we found that E2-25K SUMOylation occurred only in the ipsilateral hemispheres of WT mice, but not in the contralateral region (Figure 3e). In compliance with the ischemic damage, activated caspase-3 was observed in the ipsilateral hemispheres. We further divided the ipsilateral hemispheres after MCAO/R into three regions: core (the region most severely damaged by I/R), penumbra (a rim of the region surrounding the core), and region A (the remaining region unaffected by I/R). Western blot analysis revealed that E2-25K SUMOylation evidently occurred in the core region, paralleling caspase-3 activation (Figure 3f). These *in vivo* results imply the possibility that E2-25K SUMOylation occurs in the affected brain, probably to facilitate I/R-induced injury.

### SUMOylated E2-25K impairs proteasome activity for cell death

**Figure 4:**
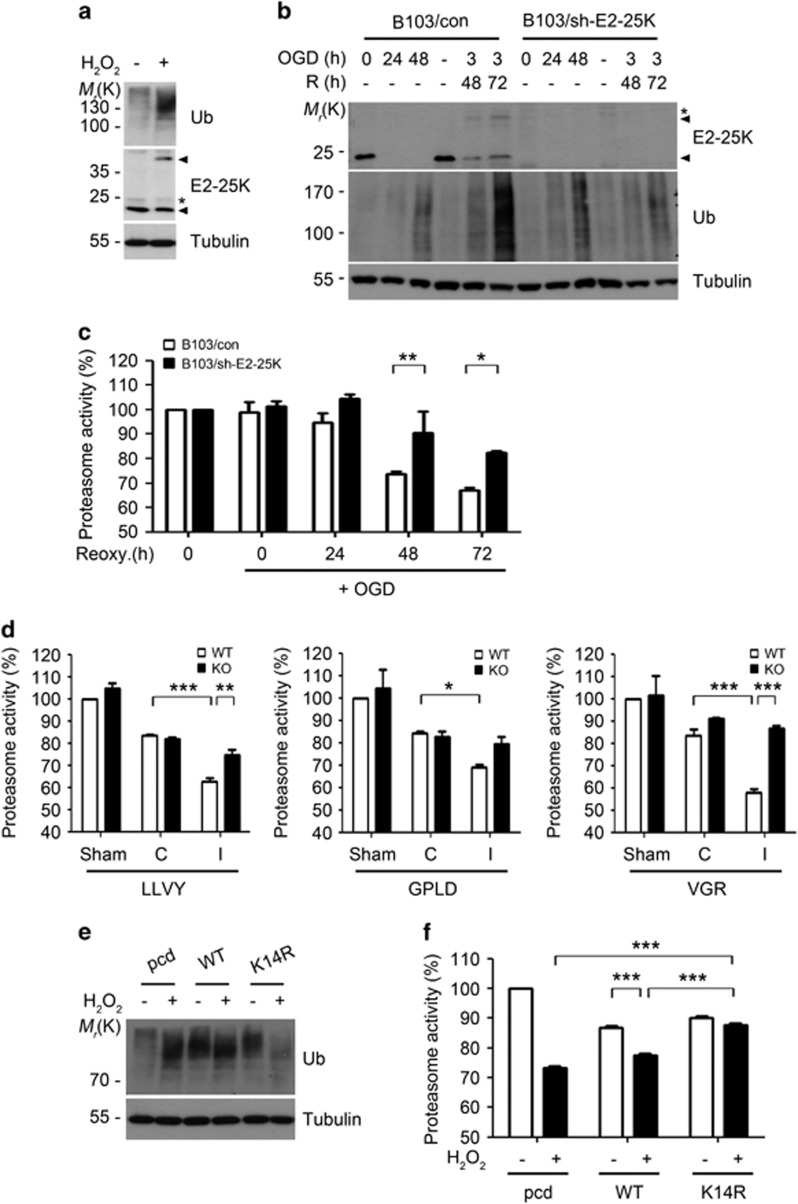
E2-25K SUMOylation under I/R condition impairs proteasome activity. (**a** and **b**) SH-SY5Y cells were treated with 100 *μ*M H_2_O_2_ for 12 h (**a**) and B103/con and B103/sh-E2-25K cells were exposed to OGD alone or followed by reoxygenation for the indicated times (**b**). Cell extracts were analyzed by western blotting. (**c**) B103/con and B103/sh-E2-25K cells were exposed to OGD for 3 h and reoxygenation for the indicated times. Cell lysates were analyzed for the proteasome activity using suc-LLVY-AMC. Values represent mean±S.E.M. (*n*=3, two-way ANOVA followed by Bonferroni's *post hoc* test, *P<0.05, **P<0.01). (**d**) E2-25K WT and KO mice were perfused for 24 h after MCAO for 30 min. Tissue lysates (except cerebellum) from non-ischemic (contralateral, C) and ischemic (ipsilateral, I) hemispheres of mouse brains were examined for chymotrypsin-like (Suc-LLVY-AMC), caspase-like (Ac-GPLD-AMC) and trypsin-like (Bz-VGR-AMC) activities of the proteasome (mean±S.E.M., sham WT and KO mice, *n*=3; MCAO WT mice, *n*=3; KO mice, *n*=4, two-way ANOVA followed by Bonferroni's *post hoc* test, *P<0.05 **P<0.01, ***P<0.001). (**e** and **f**) SH-SY5Y cells were transfected with pcDNA (pcd), E2-25K WT or K14R, treated with 100 *μ*M H_2_O_2_ for 12–20 h and analyzed by western blotting (**e**) or examined for proteasome activity using suc-LLVY-AMC (**f**). Bars represent mean±S.E.M. (*n*=3, two-way ANOVA followed by Bonferroni's *post hoc* test, ***P<0.001)

Because E2-25K has the capability to inhibit proteasome activity^27, 29, 34, 35^ and cerebral I/R decreases proteasome activity,^23^ we addressed whether E2-25K has a role in oxidative stress and I/R-mediated inhibition of proteasome activity. As expected, the accumulation of ubiquitin conjugates, an indicator of proteasome activity, and E2-25K SUMOylation were observed in SH-SY5Y cells treated with H_2_O_2_ (Figure 4a) and B103 cells incubated under OGD/R (Figure 4b). On the other hand, these effects were alleviated by E2-25K deficiency (Figure 4b). In addition, measurement of proteasome activity using a fluorogenic peptide substrate revealed that the chymotrypsin-like activity was sharply reduced in control cells during the period of OGD/R, whereas it was less decreased in B103/sh-E2-25K cells (Figure 4c). In support of these facts, analysis of *in vivo* ischemic stroke revealed that activities of all three enzymes (chymotrypsin-, trypsin- and caspase-like activities) of the proteasome were reduced in WT mice (Figure 4d). In contrast, these activities were not much impaired in E2-25K KO mice (Figure 4d). Thus, E2-25K functions to inhibit proteasome activity in cells and mouse brain during I/R. Moreover, treatment with low dose of MG132, a proteasome inhibitor, potentiated H_2_O_2_-mediated cell death (Supplementary Figure S3a), confirming that proteasome inhibition is accountable for oxidative stress-induced cell death.

To address whether E2-25K SUMOylation affected proteasome activity, we utilized the E2-25K K14R. As observed in E2-25K WT, proteasome activity was also inhibited by the ectopic expression of E2-25K K14R in control cells (Figures 4e and f). On the contrary, E2-25K K14R rescued proteasome inhibition and the accumulation of ubiquitin conjugates under oxidative stress (Figures 4e and f). Confirming this, we found that proteasome activities were equally reduced in B103/sh-E2-25K cells that were reconstituted with E2-25K WT or K14R under normoxia (Supplementary Figure S3b, upper), but were significantly rescued in B103/sh-E2-25K cells reconstituted with E2-25K K14R under oxidative stress (Supplementary Figure S3b, lower). Further, ubc9 knockdown restored E2-25K-mediated inhibition of proteasome activity to a level similar to the E2-25K K14R (Supplementary Figure S3c). Therefore, SUMOylated E2-25K functions to inhibit proteasome activity under oxidative stress.

Previous studies have shown that E2-25K synthesizes unanchored polyubiquitin chains without E3.^36, 37^ Furthermore, accrual of unanchored polyubiquitin chains is probably repressor, as it functions as a competitive inhibitor of substrate binding to the proteasome and other ubiquitin receptors.^34, 38^ Therefore, we investigated whether SUMOylated E2-25K affected polyubiquitin chain synthesis *in vitro*. There was no difference between E2-25K WT and K14R proteins in their activities to synthesize polyubiquitin chains (Supplementary Figure S3d). We again confirmed SUMOylation of E2-25K at Lys14 *in vitro* (Supplementary Figure S3e) and utilized this form in an *in vitro* ubiquitination assay. Compared with SUMOylation-defective E2-25K K14R, it seemed that SUMOylated E2-25K facilitated the formation of high-molecular-weight (HMW) ubiquitin chains (Supplementary Figure S3f). Collectively, SUMOylated E2-25K might prompt the formation of HMW ubiquitin chains under oxidative stress.

### SUMOylated E2-25K downregulates ubiquitin receptor S5a

**Figure 5:**
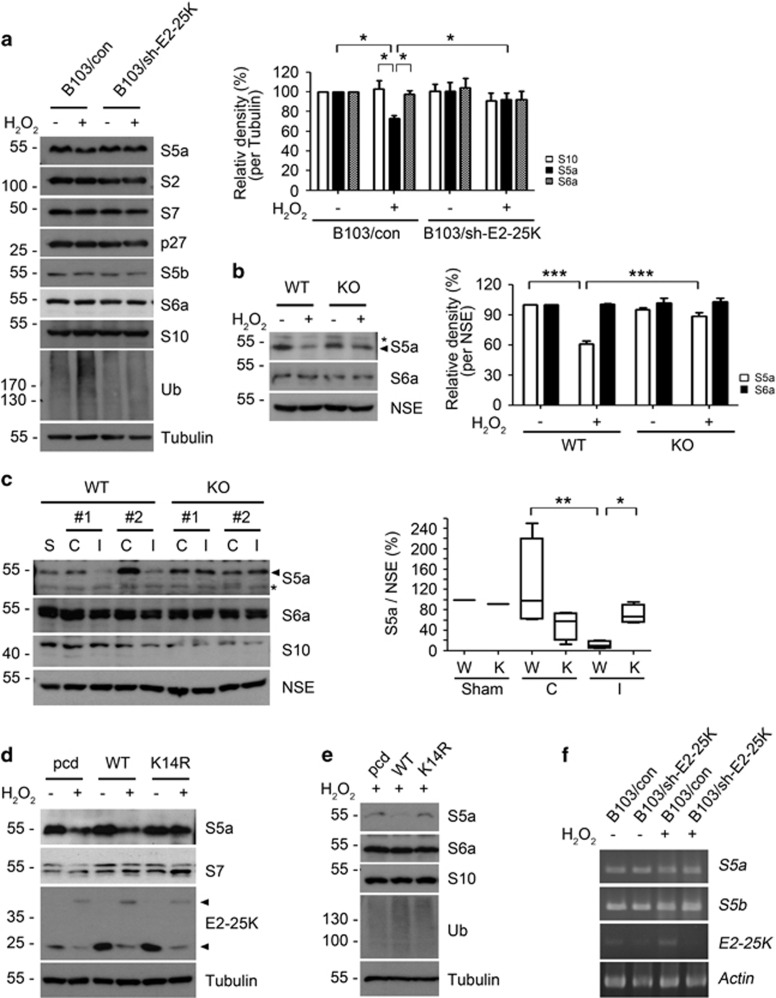
S5a within proteasome is decreased by SUMOylated E2-25K. (**a** and **b**) B103/con and B103/sh-E2-25K cells (**a**) and E2-25K WT and KO mouse cortical neurons (DIV-20) (**b**) were treated with 100–150 *μ*M H_2_O_2_ for 14–16 h and analyzed by western blotting (**a** and **b**; left). The signals of S5a on the blots were quantified by densitometric analysis and normalized by tubulin (**a**, right) or NSE (**b**, right). Bars represent mean±S.E.M., *n*=4, two-way ANOVA followed by Bonferroni's *post hoc* test, *P<0.05, ***P<0.001). (**c**) The 3–4-month-old male E2-25K WT and KO mice were perfused for 24 h after MCAO for 30 min. Tissue extracts (except cerebellum) from contralateral (C), ipsilateral (I) and sham control (S) hemispheres were analyzed by western blotting (left). The signals of S5a on the blots were quantified by densitometric analysis and normalized by NSE (mean±S.E.M., *n*=3, two-way ANOVA followed by Bonferroni's *post hoc* test, *P<0.05, **P<0.01). (**d** and **e**) SH-SY5Y (**d**) and B103/sh-E2-25K (**e**) cells were transfected with pcDNA (pcd), E2-25K WT or K14R and treated with 200 (**d**) or 70 *μ*M (**e**) H_2_O_2_ for 20 h. (**f**) B103/con and B103/sh-E2-25K cells were treated with 200 *μ*M H_2_O_2_ for 14 h. Total mRNA was isolated and subjected to RT-PCR analysis

To address the mechanism by which SUMOylated E2-25K impairs proteasome activity, we evaluated the levels of proteasome subunits under oxidative stress. Among proteasome subunits and assembly chaperones, only S5a, an ubiquitin receptor within the proteasome,^39, 40^ was significantly reduced by H_2_O_2_ treatment in B103 cells but not in B103/sh-E2-25K cells (Figure 5a). S6a, another ubiquitin receptor within the proteasome,^41^ was not affected by H_2_O_2_. Similar results were observed in primary cortical neurons; S5a, but not S6a, was reduced in an E2-25K-dependent manner under oxidative stress (Figure 5b). Furthermore, S5a was greatly reduced in the ipsilateral hemispheres of WT mice during MCAO/R (Figure 5c). In contrast, the downregulation of S5a was not observed in E2-25K KO mice. Thus, E2-25K is required for the downregulation of S5a during oxidative stress and MCAO/R.

We next examined the effect of E2-25K K14R on the regulation of S5a. Unlike E2-25K WT, ectopic expression of E2-25K K14R blocked the decline of S5a in H_2_O_2_-treated cells (Figure 5d). In normal condition, E2-25K WT and K14R had no different effects on S5a levels. Likewise, while S5a remained high in B103/sh-E2-25K cells even under oxidative stress, it was reduced in B103/sh-E2-25K cells reconstituted with E2-25K WT, but not with E2-25K K14R (Figure 5e). Accordingly, the accumulation of ubiquitin conjugates showed an inverse relationship with S5a levels. We found that this regulation of S5a had no relevance to its mRNA levels (Figure 5f). These results suggest that E2-25K SUMOylation is critical for the downregulation of S5a during oxidative stress.

We then assessed how S5a levels were altered by I/R injury. Western blotting showed that S5a was cleaved to generate a cleavage product with 36 kDa only in WT mice during OGD/R (Supplementary Figure S4a). This cleavage of S5a was evident in the core region and partial in the penumbra region but was not observed in the contralateral region (Supplementary Figure S4b). Moreover, the cleavage was proportional to E2-25K SUMOylation. As E2-25K does not have peptidase activity, S5a could be cleaved by another protease. Based on a previous report showing that S5a is cleaved by calpain during oxidative stress,^42^ we tested the effect of calpeptin, a calpain inhibitor, on S5a cleavage. As expected, S5a cleavage occurred in primary cortical neurons during oxidative stress, but was inhibited by the treatment with calpeptin (Supplementary Figure S4c). We also confirmed that intracellular Ca^2+^ level was upregulated in WT mouse cortical neurons, but less in E2-25K KO neurons, following H_2_O_2_ treatment or under OGD/R (data not shown). Thus, it is likely that S5a is cleaved by calpain, which is activated by E2-25K during MCAO/R and H_2_O_2_ treatment.

### Altered S5a dissembles the lid in the 19S and impairs the activity of 26S proteasomes

**Figure 6:**
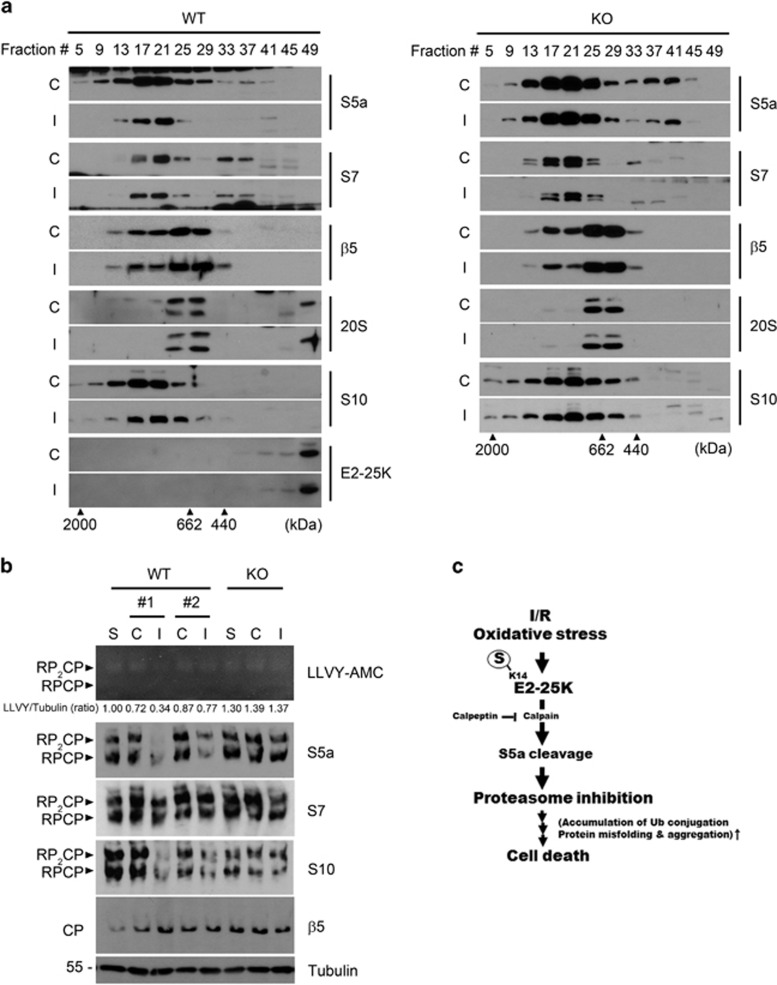
E2-25K deficiency rescues the impaired activity of 26S proteasome during MCAO/R. (**a**) The 3–4-month-old male E2-25K WT (left) and KO (right) mice were subjected to MCAO/R as in Figure 5c and brain extracts were fractionated by chromatography analysis using a Superose 6 column. The fractions were analyzed by western blotting using the indicated antibodies against proteasome subunits. Blue dextran (2000 kDa), thyroglobulin (662 kDa) and ferritin (440 kDa) were used as molecular weight markers. (**b**) The same brain extracts were analyzed by native gel overlaid with Suc-LLVY-AMC (top) and subsequently by western blotting (middle). The same lysates were subjected to SDS-PAGE and western blotting (bottom). Tubulin served as an internal control and relative ratios of the proteasome activity to the level of tubulin (LLVY/tubulin, ratio) were also determined. CP, core particle; RP, regulatory particle. (**c**) Schematic diagram showing the proposed role of SUMOylated E2-25K in tissue damage during I/R and oxidative stress

S5a, which was identified as a subunit of the 19S regulatory particle of the 26S proteasome,^39^ is located at the interface between the lid and base of 19S.^43, 44^ To address how the reduction of S5a impairs proteasome activity, we fractionated tissue extracts of the ipsilateral hemisphere with FPLC gel filtration analysis. Western blotting of the fractions showed that S5a in the 26S proteasome fractions (#9–17) and subparticle fractions (#25–29) was significantly reduced in WT ipsilateral hemisphere as compared with contralateral region, whereas other proteasome subunits, such as S7, *β*5, and 20S, were not altered (Figure 6a, left). In contrast, such reduction of S5a was not observed in E2-25K KO mice (Figure 6a, right). Interestingly, S10, one of the 19S lid subunits, was not altered in their total amount during oxidative stress (Figures 5a, c and e), but was reduced in the 26S proteasome fractions (#9–17) and shifted towards low-molecular-weight fractions (Figure 6a, left). Again, these changes of S10 were not observed in E2-25K KO mice (Figure 6a, right). Thus, reduced S5a in the 26S and 30S proteasome may cause disassembly of the lid in the 19S of the 26S proteasome.

We further examined the disassembly and activities of proteasome complexes with native gel analysis. The results revealed that a significant loss of enzyme activities in the 26S (RP-CP) and 30S (RP_2_-CP) proteasomes was observed in WT mice but not in E2-25K KO mice (Figure 6b, top). Also, S5a levels were much less in the 26S (RP-CP) and 30S (RP_2_-CP) proteasomes of the ischemic hemispheres in WT mice (0.34 and 0.77, compared with untreated control) than in E2-25K KO mice (1.37, compared with untreated control; Figure 6b). Like S5a, S10 levels were also decreased in the 26S and 30S proteasomes of WT ipsilateral hemisphere. Consistently, other subunits, such as S7 in the base of 19S and *β*5 in the 20S, were not altered in these complex. In addition, detailed analysis of every FPLC fractions confirmed drastic difference in the levels of S5a of the 19S (fractions #21–24) between the ischemic hemispheres of WT and E2-25K KO mice (Supplementary Figure S5). Collectively, these observations indicate that the level of S5a is downregulated by E2-25K under MCAO/R to alter the complex and activity of proteasomes, leading to proteasome impairment.

## Discussion

Post-translational modification by SUMO is involved in various cellular processes and SUMOylated proteins in these processes can alter protein–protein interactions, subcellular localization and protein stability, or change their activities.^45, 46, 47^ In the present study, E2-25K SUMOylation occurs under OGD/R, but not by OGD alone, and by the pathophysiological concentrations of H_2_O_2_ (100–300 *μ*M). As ROS produced under OGD/R is greater than that under OGD,^48^ it seems that ROS levels are critical for E2-25K SUMOylation. Interestingly, E2-25K was not SUMOylated in cells that were exposed to low doses (1 or 2 *μ*M) of H_2_O_2_,^29^ as well as high doses of >1 mM H_2_O_2_. Rather, E2-25K was induced without SUMOylation under those conditions (Supplementary Figure S1k) and functions to modify caspase-12.^29^ Thus, a certain level of intracellular ROS is required for E2-25K SUMOylation. Considering that E2-25K functions in diverse pathways, these results imply that E2-25K has multiple roles depending on its modification and expression level. In fact, ROS level has been measured in many diseases;^49^ it is ~160 *μ*M in cerebral I/R injury^50^ and is upregulated by the A*β* peptide by up to three-fold in Alzheimer's disease.^51^

The issue of whether inhibition of proteasome activity has a protective or detrimental role in stroke is controversial. With respect to the immunoproteasome, proteasome inhibitors are known to prevent ischemic damage.^52, 53^ Although their mechanisms of action are unknown, proteasome inhibitors have been tested as neuroprotective drugs. On the other hand, proteasome dysfunction causes protein aggregation and neuronal cell death after cerebral I/R.^22, 24, 25, 54^ Even the immunoproteasome blocks protein aggregation and preserves protein homeostasis upon oxidative stress associated with inflammation.^55, 56^ Our data are in line with the latter in that impaired proteasome activity is harmful in the context of I/R injury. In this case, an important question of how E2-25K impairs proteasome activity remains. E2-25K synthesizes unanchored polyubiquitin chains without E3^57, 58, 59^ and proteasome activity can be hampered by them.^34, 38^ In the same context, our analysis utilizing E2-25K K14R suggests that E2-25K SUMOylation stimulates the synthesis of polyubiquitin chains and restrains proteasome activity under oxidative stress. Nonetheless, E2-25K K14R still synthesizes polyubiquitin chains as much as E2-25K WT but much less than SUMOylated E2-25K, implying that the regulating E2-25K expression is also another way to synthesize polyubiquitin chains. A detailed characterization of the difference between these activities remains to be resolved.

A previous report showed that E2-25K is SUMOylated at K14 *in vitro* and this SUMOylation interferes with the transfer of ubiquitin from the E1 enzyme to E2-25K.^34^ However, in our study, SUMOylated E2-25K was better at generating polyubiquitin chains than E2-25K K14R. Moreover, E2-25K was SUMOylated *in vitro* despite the presence of E1 (data not shown). This possibly occurs due to the presence of ubiquitin-charged E2-25K. In fact, there is no difference between ubiquitin-charged deSUMOylated and SUMOylated E2-25K in the ability to synthesize diubiquitin, that is, a facility of ubiquitin transfer.^34^ If E2-25K is able to accept activated ubiquitin from not only E1 but also from E2-25K through interaction with another ubiquitin-charged E2-25K as an ubiquitin donor,^60^ SUMOylated E2-25K may continue to generating polyubiquitin chains.

Importantly, S5a was downregulated in affected neurons and tissues under oxidative stress and specifically reduced in the 26S and 30S proteasome complexes. Deletion of S5a results in difficulties in protein degradation.^61, 62^ Thus, the lack of S5a in the proteasome causes accumulation of ubiquitin conjugates. Although previous reports have shown that oxidative stress leads to the separation of the 20S and 19S in neuronal cells,^63, 64^ downregulation of S5a did not affect the dissociation 20S and 19S in the MCAO/R mice in our study. Recently, rpn13, another polyubiquitin receptor in the proteasome, has also been reported to have a redundant role in the recognition of ubiquitinated proteins in a Drosophila.^65^ Unlike rpn13, however, only S5a can connect the base and lid and stabilize them within 19S.^66, 67, 68^ Thus, according to our results showing the loss of S10 from proteasome following S5a reduction, impaired proteasome activity under oxidative stress is likely due to disassembly of the lid within 19S from 26S proteasome. Indeed, proteasome complex lacking the lid was observed in S5a-deficient cells.^69, 70^ In this process, we speculate that the synthesis of polyubiquitin chains by SUMOylated E2-25K may form a positive-feedback loop to further impair proteasome activity. Eventually, the reason for the importance of impaired proteasomes in tissue damage remains to be assessed in the future.

In conclusion, our study suggests that E2-25K is an arbitrator of I/R-induced damage and its SUMOylation confers susceptibility to I/R by impairing proteasome activity, thus providing a potential therapeutic target in stroke.

## Materials and methods

### Cell culture and DNA transfection

B103 and SH-SY5Y cells were maintained in Dulbecco's modified Eagles medium (DMEM; Invitrogen, Carlsbad, CA, USA) containing 10% fetal bovine and penicillin/streptomycin. Primary neurons were prepared as described previously.^28^ In brief, the cortical neurons were cultured from embryonic day 14 and incubated in neurobasal media (GIBCO BRL, Carlsbad, CA, USA) containing B27 (Invitrogen). Transfection was carried out with polyethylenimine or LipofectAMINE reagent (Invitrogen) following the manufacturer's instructions. For OGD/reoxygenation, cells were exposed to glucose-free DMEM within a hypoxic chamber (Billups-Rothenberg, Inc, San Diego, CA, USA, 1% O_2_). After OGD, the cells were reoxygenated under normoxic conditions in normal DMEM.

### Antibodies

For western blotting, the following antibodies were used: anti-GFP, anti-tubulin, anti-Ub, anti-E2-25K, anti-synaptophysin antibodies (Santa Cruz Biotechnology, Santa Cruz, CA, USA); anti-caspase-3, anti-S5a antibodies (Cell Signaling, Beverly, MA, USA); anti-SUMO1, anti-NSE antibodies (Zymed, Carlsbad, CA, USA); anti-S10 antibody (Genetex, Irvine, CA, USA); anti-S5b antibody (Novus, Littleton, CO, USA); anti-S5a, anti-S7, anti-PAC2, anti-*β*5, anti-20S core antibodies (Biomol, Farmingdale, NY, USA); anti-S2 antibody (Abcam, Cambridge, UK); anti-p27 antibody (Sigma-Aldrich, St. Louis, MO, USA); and anti-S6a antibody (Enzo, Plymouth Meeting, MA, USA). Anti-E2-25K antibody was described previously.^28^

### Plasmid construction

The E2-25K and its active site mutant (C92S) have been described previously.^28^ E2-25K K14R was generated by PCR using primers containing the corresponding mutation (E2-25K [K14R]-5′, 5′-CGGGAGTTCAGAGAGGTGCTG-3′ E2-25K [K14R]-3′, 5′-CAGCACCTCTCGAACTCCCG-3′), and confirmed by DNA sequencing analysis. To construct the E2-25K shRNA, heteroduplex oligomers containing sequences of human or rat E2-25K (E2-25K [shRNA#1]-5′, 5′-GATCCCCAAGCAGACAGCTCGACTTTTTCAAGAGAAAAGTCGAGCTGTCTGCTTTTTTTA-3′ E2-25K[shRNA#1]-3′, 5′-AGCTTAAAAGCAGACAGCTCGACTTTTCTCTTGAAAAAGTCGAGCTGTCTGCTTGGG-3′ (E2-25K [shRNA#2]-5′, 5′-GATCCCCGAATCAAGCGGGAGTTCAATTCAAGAGATTGAACTCCCGCTTGATTCTTTTTA-3′ E2-25K[shRNA#2]-3′, 5′-AGCTTAAAAAGAATCAAGCGGGAGTTCAATCTCTTGAATTGAACTCCCGCTTGATTCGGG-3′) were synthesized, annealed and cloned into the *Bgl*II and *Hin*dIII sites of pSuper-Neo (OligoEngine, Seattle, WA, USA). For construction of His-E2-25K was subcloned into pET-28a using the primers (E2-25K-Hisx6-5′-*Eco*RI, 5′-CCGGAATTCCGGATGGCCAACATCGCGGTG-3′ E2-25K-Hisx6-3′-*Xho*I, 5′-CCGCTCGAGCGGTCAGTTACTCAGAAGCAA-3′).

### Generation of stable cell line

B103 and SH-SY5Y cells were transfected with E2-25K or E2-25K shRNA using LipofectAMINE reagent for 24 h and then grown in selection medium containing 2 mg/ml of G418 (Invitrogen) for 2 weeks to generate stable E2-25K overexpression or knockdown cells. After single-cell cloning, the clones were screened by western blot analysis.

### Viability test

Cell viability was measured using the propidium iodide. In the transient transfection experiments, cell viability was determined based on the morphology of GFP-positive cells under a fluorescence microscope (Olympus, Tokyo, Japan) and by trypan blue exclusion assays as described.^28^

### E2-25K KO mice

Maintenance of and experimentation with E2-25K WT and KO mice on a BALB/c background^29^ were performed in accordance with the animal care guidelines of Seoul National University and the ARRIVE guidelines,^71^ including randomization, blinding, appropriate controls, inclusion and exclusion criteria, reporting of all animals used, and so on. All mice described for experiments in this study have been produced through intercross breeding between heterozygous E2-25K KO mice.

### MCAO

The 3–4-month-old male E2-25K WT or KO mice were anesthetized with intraperitoneal injection of Zoletil/Rompun mix, and transient MCAO was performed as previously described.^72^ In brief, the right common carotid artery (CCA) was exposed through a midline incision in the neck and unilateral MCAO was performed by inserting a 6-0 silicone rubber-coated monofilament (Doccol Corp., Sharon, MA, USA). The suture was inserted into the CCA and proceeded to the internal carotid artery. The MCA was occluded for 30 min and then the suture was withdrawn to allow 24 h of reperfusion before sacrifice. In sham-operated mice, the carotid arteries were prepared surgically but the filament was not inserted. Then, the brain was collected and placed in a metallic brain matrix for tissue slicing (Daejong, Seoul, Korea). Coronal slices (2 mm from the olfactory bulb) were incubated in 2% TTC for 20 min in PBS at 37 °C, and then fixed with 4% paraformaldehyde. The stained slices were scanned and subsequently measured for the surface area of the slices and the ischemic lesion using FujiFilm Multi-Gauge 3.0 software (FujiFilm Medical Systems, Hanover Park, IL, USA). The neurobehavioral deficits were measured as previously described.^73^ In brief, each mouse was assigned a score of 0–4: 0=no observable neurological deficit; 1=failure to extend contralateral forepaw; 2=circling in a direction contralateral to infarct; 3=falling in a direction contralateral to infarct; and 4=no spontaneous movement. In the transient MCAO group, a hanging wire grip test was also performed following the neurological score assessment.^74^ In brief, mice were put on top of a wire cage lid that was shaken gently. The wire cage lid then was inverted. The elapsed time until the mouse fell was recorded three times.

### IP assay

HEK293T cells lyzed in lysis buffer (20 mM Tris-HCl (pH 7.4), 150 mM NaCl, 10 mM *N*-ethylmaleimide, 1% Triton X-100 and protease inhibitor cocktail). Cell lysates were centrifuged at 12 000 r.p.m. for 20 min at 4 °C. Supernatant was incubated with anti-GFP antibody overnight at 4 °C and pulled down by protein G Sepharose beads (GE Healthcare, Fairfield, CT, USA).

### Proteasome activity assays

After preparation of cell lysates in retic buffer, proteasome activities were measured using the fluorogenic substrates Suc-LLVY-AMC, Bz-VGR-AMC and Ac-GPLD-AMC (Biomol) and a fluorometer (EnVision Multilabel Reader; PerkinElmer, Waltham, MA, USA) with excitation at 380 nm and emission at 460 nm.

### *In vitro* ubiquitination and SUMOylation

*In vitro* enzyme reactions was performed in a buffer containing purified 10 *μ*g 6xHis-E2-25K, 5 mM MgCl_2_, 4 mM ATP, 10 mM creatine phosphate and 10 U creatine phosphokinase. For polyubiquitin chain formation assay, 15 *μ*g ubiquitin (Boston Biochem, Cambridge, MA, USA) and 500 ng 6xHis-E1 (Boston Biochem) were added to the above mentioned mixture and then incubated at 37 °C for 6 h. For SUMOylation assay, 5 *μ*g His-Ubc9 (Sino Biological Inc., Beijing, China), purified 10 *μ*g GST-SUMO1 (instead of Ub) and 1 *μ*g SUMO-E1 (Boston Biochem) were used. The reaction were incubated at 37 °C for 2 h.

### Reverse transcriptase-PCR

Total RNA was purified with TRIzol reagent (Invitrogen) and used for reverse transcription as described previously.^75^ PCR was performed for 15–25 cycles using following synthetic oligonucleotides sets: E2-25K RT-5′ (5′-GCGAATTCTATGGCAACATCGCGGTG-3′); E2-25K RT-3′ (5′-GGGGTACCCCGTTACTCAGAAGCAG-3′); S5a RT-5′ (5′-CTGCAGGCCCAGGAT-3′); S5a-RT-3′ (5′-GGCACCAAGACCCAGCAT-3′); actin RT-5′ (5′-TGAGAGGGAAATCGTGCGTG-3′); actin RT-3′ (5′-TGCTTGCTGATCCACATCTGC-3′).

### Native PAGE

Native gel analysis was performed as previously described.^76^ Cell lysates were separated on 4% (w/v) native PAGE at 4 °C. The gel was overlaid with buffer containing Suc-LLVY-AMC and transferred to nitrocellulose membranes for western blot analysis.

### Preparation of cell extracts and separation of proteasome complexes by gel filtration

Gel filtration was performed as previously described.^77^ In brief, brain extracts were filtered through a 0.2-*μ*m membrane (Sartorius, Goettingen, Germany). Gel filtration was carried out using a Superose 6 FPLC column (AKTA; GE Healthcare), and 0.25-ml fractions were collected.^33^
