# Supplementary material for: E2-25K SUMOylation inhibits proteasome for cell death during cerebral ischemia/reperfusion
Source: Cell Death Dis. 2016 Dec 29;7(12):e2573–. doi: 10.1038/cddis.2016.428 (PMC5261013; doi:10.1038/cddis.2016.428)
Supplement: Supplementary Figure Legends [file cddis2016428x1.docx]

**Supplementary Data**

**Supplementary Figure S1. E2-25K mediates neuronal cell death and is SUMOylated during oxidative stress.** (**a**) Expression level of E2-25K in B103 wild-type (B103/con) and E2-25K knockdown (B103/sh-E2-25K) cells. (**b**-**d**) B103/con and B103/sh-E2-25K cells were exposed to OGD for 3 h followed by reoxygenation for the indicated times and observed under bright field microscope (**b**). Cell death rates were determined after staining with PI. Values represent mean ± S.E.M. (*n* = 3, two-way ANOVA followed by Bonferroni’s *post-hoc* test, ****P* < 0.001) (**c**). Cell extracts were analyzed with western blotting (**d**). (**e** and **f**) SH-SY5Y/con, SH-SY5Y/E2-25K and SH-SY5Y/sh-E2-25K cells were treated for 3 h with OGD followed by reoxygenation for the indicated times (**e**) or exposed to 200 μM H_2_O_2_ for 21 h (**f**). Expression levels of E2-25K were examined by western blotting (**e**, *insert*). (**g**) E2-25K WT and KO mouse cortical neurons (DIV-20) were treated with 100 μM H_2_O_2_ for 16 h**.** Cell death rates were determined after PI staining. Values represent mean ± S.E.M. (*n* = 3, two-way ANOVA followed by Bonferroni’s *post-hoc* test, ***P* < 0.01, ****P* < 0.001) (**e**-**g**). (**h**) B103 cells were incubated with the indicated concentrations of H_2_O_2_ for 12 h in the presence or absence of preincubation with 500 μM NAC for 1 h and analyzed by western blotting. (**i**) SH-SY5Y cells were exposed to OGD for 3 h followed by reoxygenation for either 50 h or 70 h, or treated with 100 μM (*left*) or 200 μM (*right*) H_2_O_2_ for 24 h. Cells were assessed for ROS levels by FACS using H_2_DCFDA. (**j**) B103/con and B103/sh-E2-25K cells were treated with the indicated concentrations of H_2_O_2_ for 15 h and analyzed by western blotting. (**k**) SH-SY5Y cells were treated with the increasing concentrations of H_2_O_2_ in growth media for 16 h or with 1 or 2 μM H_2_O_2_ in serum-free media for 48 h. Cell extracts were analyzed by western blotting. Arrow heads and asterisks indicate E2-25K and non-specific signals, respectively.

**Supplementary Figure S2. Expression of SUMOylation-defective E2-25K K14R inhibits H_2_O_2_-induced cell death.** SH-SY5Y cells were transfected with pcDNA (pcd), E2-25K WT or mutants (K14R, C92S) for 24 h and then treated with 150 μM H_2_O_2_ for 9 h. Cell viability was assessed by trypan blue exclusion assay (mean ± S.E.M., *n* = 3, one-way ANOVA followed by Tukey’s *post-hoc test*, **P* < 0.05, ***P* < 0.005).

**Supplementary Figure S3. SUMOylation-defective E2-25K K14R rescues the impaired proteasome activity under oxidative stress.** (**a**) SH-SY5Y cells were incubated with indicated concentrations of H_2_O_2_ for 16 h in the presence or absence of 0.1 (+) or 0.2 μM (++) MG132. Cell death rates were determined after staining with PI. Bars represent mean ± SD (*n* = 3). **P* < 0.05, ***P* < 0.005, ****P* < 0.0005, *t* test. (**b**) B103/sh-E2-25K cells were transfected with pcDNA (pcd), E2-25K WT or its mutant and then left untreated (*upper*) or treated with (*lower*) 200 μM H_2_O_2_ for 16 h. Chymotrypsin-like activity was measured using suc-LLVY-AMC (*left*). Bars represent mean ± SD (*n* = 3). **P* < 0.01, #*P* < 0.0001, *t* test. The expression levels of E2-25K and GFP were examined by western blotting (*right*). (**c**) SH-SY5Y cells were transfected with pcDNA (pcd), E2-25K WT, KR mutant or together with shRNAubc9, and then exposed to 150 mM H_2_O_2_ for 14 h. Cell lysates were analyzed for proteasome activity using suc-LLVY-AMC. Bars represent mean ± S.E.M. (*n* = 3, one-way ANOVA followed by Tukey’s *post-hoc* test, ****P* < 0.001). (**d**) *In vitro* polyubiquitin chain formation assay was performed with E1 (500 ng), ubiquitin (Ub, 15 μg) and E2-25K (10 μg) proteins at 37°C for 6 h and the reaction products were analyzed by western blotting (*top* and *middle*) or the protein gel was stained by Coomassie blue (*bottom*). (**e**) *In vitro* SUMOylation assay was performed with purified His-E2-25K WT or K14R (1 μg), SUMO-E1 (200 ng), GST-SUMO1 (10 μg) and His-Ubc9 (5 μg) proteins in the presence of ATP (4 mM) for 2 h. The reaction products were analyzed by western blotting (*upper*) and the protein gel was stained with Coomassie blue (*lower*). Arrow heads and asterisks indicate E2-25K and non-specific signals, respectively. (**f**) *In vitro* polyubiquitin chain formation assay was performed for 6 h by adding ubiquitin (Ub, 15 μg) and E1 (500 ng) proteins into the reactions described in (**e**). The reaction mixtures were analyzed by western blotting (*top* and *middle*) and the protein gel was stained with Coomassie blue (*bottom*).

**Supplementary Figure S4. E2-25K is required for S5a cleavage by calpain under oxidative stress.** (**a**) Brain extracts prepared in Figure 5c were analyzed by western blotting. (**b**) Tissue extracts of ischemic core, penumbra and region A in the ipsilateral region of MCAO/R-treated WT mouse brain were examined by western blotting. (**c**) WT mouse cortical neurons (DIV-9) were pretreated with calpeptin for 2 h and then were exposed to 150 μM H_2_O_2_ for 14 h. Arrow heads and asterisks indicate E2-25K and non-specific signals, respectively.

**Supplementary Figure S5. E2-25K decreases S5a in 26 and 30S proteasome.** The every fractions (#5–28) of WT and E2-25K KO brain extracts described in Figure Figure 46a were analyzed by western blotting using antibodies against proteasome subunits.
